# The Arabic Classroom Participation Questionnaire: Validation with d/Deaf and Hard of Hearing University Students

**DOI:** 10.3390/bs16101792

**Published:** 2026-10-01

**Authors:** Faisl M. Alqraini, Khalid N. Alasim

**Affiliations:** Department of Special Education, Prince Sattam bin Abdulaziz University, Al-Kharj 11942, Saudi Arabia; k.alasim@psau.edu.sa

**Keywords:** d/Deaf and hard of hearing, classroom participation, communication access, scale adaptation, university students

## Abstract

The Classroom Participation Questionnaire (CPQ) was developed with school-aged d/Deaf and hard of hearing (d/Dhh) students, but no Arabic version exists for university populations. This study adapted the CPQ into Arabic, extended it to d/Dhh university students, and examined its internal structure, reliability, and the associations of communication understanding with communication-related positive and negative affect. In total, 135 d/Dhh students at Saudi public universities completed the 26-item Arabic CPQ. Internal consistency was excellent (*α* = 0.91–0.96). Positive- and negative-affect items formed clear, separate factors, whereas instructor- and peer-understanding items were highly interrelated (*r* = 0.83) and were combined into one composite; a four-factor confirmatory model showed acceptable fit (CFI = 0.90, RMSEA = 0.089). Communication understanding was strongly associated with positive affect (*r* = 0.69) and weakly with negative affect (*r* = 0.24), and the two affective dimensions were largely independent (*r* = 0.16). Female students reported more positive and less negative affect; hearing-device use added no independent association. The Arabic CPQ functioned reliably in this population. Because the understanding and positive-affect subscales share communication-specific content, their strong association partly reflects item overlap; the independence of the two affective dimensions suggests that accessibility and psychosocial support address distinct needs.

## 1. Introduction

Active involvement in the life of the classroom ranks among the strongest correlates of how well university students learn, stay enrolled, and grow both academically and personally. Scholars generally treat such involvement as a layered phenomenon operating on behavioral, cognitive, and emotional planes at once, each plane feeding into achievement and retention (Fredricks et al., 2004). What binds these planes together inside a lecture hall is communication: teaching proceeds through talk, questioning, collaborative work, and exchange with classmates, so a learner’s foothold in that communicative flow largely decides whether participation is possible at all (Stinson et al., 2022). The clarity with which students can communicate in class therefore bears directly on how engaged they become and on the texture of what they experience while learning (Antia et al., 2007; Marchetti et al., 2012).

An influential way of unpacking this involvement comes from Antia et al. (2007), who developed the Classroom Participation Questionnaire (CPQ) with school-aged d/Dhh students in Grades 4–10 and framed classroom participation as a composite of distinct perceptions: how well learners feel they grasp what instructors convey, how well they follow their classmates, and what they feel emotionally as the interaction unfolds. The first two perceptions index the cognitive face of access, the act of comprehension itself, whereas the third captures its affective face. That affective face is in turn two-sided, spanning agreeable states such as enjoyment, assurance, and a sense of fitting in, and disagreeable ones such as apprehension, irritation, and feeling shut out. Because a learner who cannot track the talk around them can neither understand nor feel at ease, communicative reach lies at the core of whether participation succeeds.

The affective layer deserves attention on its own terms rather than as a mere by-product of understanding. Studies of emotion in achievement settings show that pleasant, energizing states such as interest and enjoyment tend to broaden attention, sustain effort, and embolden learners to communicate and join in, while distress and frustration tend to narrow focus and weaken the self-regulation that participation requires (Pekrun, 2006; Pintrich, 2003). Tracing where these emotions originate thus matters to anyone working to strengthen engagement, and it matters most for students whose entry into classroom talk cannot be assumed. Throughout the manuscript, a deliberate distinction is maintained between broader emotional well-being and the narrower construct measured here, namely communication-related positive and negative affect: the situated feelings that arise while following and joining classroom talk. These situated experiences merit attention in their own right because recurring episodes of communicative comfort or frustration are plausible everyday ingredients of students’ sense of inclusion, belonging, and sustained participation in higher education, even though the CPQ itself does not measure these broader constructs.

Securing that entry is harder for students who are d/Deaf or hard of hearing (d/Dhh), because university instruction leans on speech delivered at pace and on overlapping verbal exchange (Knoors & Marschark, 2014). A hearing peer can listen while looking elsewhere; a d/Dhh student frequently cannot, and must instead spread a single channel, namely vision, across an instructor, an interpreter, on-screen captions, and slides by turns (Hardebeck et al., 2026; Mathew & Peiris, 2026). The demand intensifies in seminars and group activities, where the speaker keeps changing and the thread of conversation will not hold still (Powell et al., 2014; Stinson & Liu, 1999); under such conditions, missed exchanges, drained concentration, retreat from interaction, and a sense of standing apart have all been documented (Eichengreen et al., 2026; Stinson et al., 2022).

Access is shaped further by the room itself. Ambient noise, unhelpful acoustics, and a brisk teaching tempo each erode what reaches a d/Dhh student, especially where learning turns on open discussion (Percival et al., 2025). Individual auditory profiles add another dimension: how severe a student’s hearing loss is, and whether they wear a hearing aid or use a cochlear implant, conditions how much spoken input arrives, yet even well-fitted technology leaves the task of parsing rapid, layered speech unsolved (Hardebeck et al., 2026; Marschark et al., 2015). Where settings are welcoming and barriers stay low, by contrast, the dividend appears as firmer comprehension, warmer ties with peers, a sense of belonging, and more spirited engagement (Antia et al., 2011; Hintermair, 2008; Listman et al., 2024). For many d/Dhh students, sign language constitutes a communication profile in its own right, so classroom access depends not only on residual hearing and speech but also on whether signed communication is available and usable in the lecture setting.

The communication practices of instructors and peers can be as influential as a student’s own hearing abilities. A class grows easier to enter, and more rewarding to sit in, when instructors adopt reachable ways of speaking and arrange interaction so that all can take part, and when classmates remain attentive to communication needs and bend their own habits during shared work (Aprilia & Nisa, 2022; Paatsch et al., 2022).

Participation and its affective correlates are also patterned by gender. Among d/Dhh students, gender has been linked to differences in social participation and well-being, although the evidence is mixed (Alsamih, 2024). Because the present sample includes both men and women, gender is examined here as an additional correlate of communication-related affect, alongside hearing-device use.

Although comprehension and affect clearly move together, the way they relate for d/Dhh learners has been examined mainly with school-aged samples and largely within the original validation of the measure used here. Antia et al. (2007) established the internal-structure validity of the CPQ partly by examining the relationships among its subscales, and the understanding and positive-affect dimensions were closely associated. Because these subscales also share communication-specific content, a strong association between them is expected on measurement grounds as well as substantive ones. The present study therefore does not treat a strong understanding–positive-affect association as a novel discovery; instead, it asks how these communication-related dimensions relate to one another in a new linguistic and educational context, Arabic-speaking university students, and whether patterns observed with school-aged children extend to this population once the shared content of the subscales is acknowledged.

### 1.1. Theoretical Framework

Two lenses orient the study. The first is Vygotsky’s (1978) sociocultural account of learning, on which knowledge is assembled through socially situated exchange rather than in solitude. By this logic, a student’s capacity to join discussion, cooperate on tasks, and work with an instructor cannot be separated from their capacity to take part in the communication that carries those activities. The account bears directly on d/Dhh students, whose participation is gated by communicative reach, and it treats that reach as foundational to how classroom engagement, emotional tone included, emerges.

A second consideration concerns the nature of the affect examined here. Although achievement-emotion theory (Pekrun, 2006) describes emotions that arise around learning and achievement, such as enjoyment, pride, and anxiety, the affective dimensions of the CPQ are narrower: they capture how students feel about understanding and being understood in class, that is, communication-related affect. The present study therefore concerns communication-related positive and negative affect specifically, rather than the broader space of achievement emotions.

Positive and negative affect are widely modeled as partly separable systems rather than opposite ends of a single dimension (Cacioppo & Berntson, 1994; Watson & Tellegen, 1985). This bivalent view motivates treating communication-related positive and negative affect as distinct outcomes and examining how each relates to perceived communication access, without assuming that a single continuum links them.

Within the Saudi context, access to higher education for d/Dhh students has broadened in recent years through inclusion-oriented policy and dedicated programming, with universities providing accommodations such as sign-language interpreting, note-taking support, assistive technologies, and, increasingly, AI-enabled tools (Alkahtani, 2024; Alkharji & Loh, 2022; Alsamih, 2024). Even so, communication barriers, social isolation, and limited understanding from faculty and peers continue to affect the experiences of some d/Dhh students in Saudi universities (K. N. Alasim, 2018; K. Alasim & Paul, 2018; Alqraini & Alasim, 2021; Alsamih, 2024), underscoring the need to understand how communicative access shapes their engagement.

### 1.2. The Present Study

Although Saudi and international research has examined social participation, inclusion, communication barriers, and, separately, the cognitive and affective dimensions of classroom participation among d/Dhh students, little is known about how comprehension of classroom communication relates to the positive as opposed to the negative dimension of emotional experience, or whether it relates to them to the same degree. The present study addresses this gap by examining whether classroom communication understanding is associated with positive and negative emotional experience to differing degrees among d/Dhh students in Saudi universities.

The study was guided by three research questions: (1) To what extent is classroom communication understanding associated with the positive and negative emotional experiences tied to classroom participation? (2) Does the strength of this association differ between the positive and negative affective dimensions? (3) What is the independent contribution of gender and hearing-device use to emotional experience, over and above communication understanding? Correspondingly, it was hypothesized that communication understanding would be positively associated with positive emotional experience (H1); that its association with positive experience would be significantly stronger than its association with negative experience (H2); and that gender and hearing-device use would each account for variance in emotional experience beyond communication understanding (H3).

The analysis was deliberately confined to the variables most central to this question and best supported by the data. Classroom communication understanding, formed as a composite of understanding the instructor and understanding peers, served as the focal explanatory variable; the positive and negative dimensions of emotional experience served as the two outcomes; and gender and hearing-device use were included as additional explanatory variables, both because they are theoretically relevant to communicative access and because they were sufficiently represented in the sample to allow stable estimation. Other characteristics that might bear on participation, including degree of hearing loss treated as a separate factor, field of study, and the availability of sign-language interpretation, were not modeled, because their markedly uneven distribution in this sample, with several sparsely populated categories, would not have supported reliable comparison.

## 2. Materials and Methods

### 2.1. Research Design

This study employed a cross-sectional, descriptive–correlational quantitative design to examine how the cognitive and affective dimensions of classroom participation relate to one another among d/Dhh university students in Saudi Arabia. A correlational approach suited the aim, which was to estimate associations among variables as they naturally occur rather than to manipulate them (Creswell & Creswell, 2018).

### 2.2. Participants

Eligible participants were d/Dhh students attending public universities in Saudi Arabia. Figures released by the Authority for the Care of Persons with Disabilities (2022) put the number of disabled students across the Kingdom’s public universities at 5681, of which 710 are d/Dhh, and it was this group that defined the population of interest. Recruitment proceeded by non-probability convenience sampling. Of 142 returned questionnaires, seven were excluded because responses were missing on one or more core study variables (CPQ items or the demographic covariates); exclusion was listwise, no imputation was performed, and the 135 retained cases contained no missing item-level responses. Table 1 summarizes the sample: 63.7% were men (*n* = 86) and 36.3% women (*n* = 49). Severe hearing loss was the most common profile (65.9%), with moderate (26.7%) and mild (7.4%) loss less frequent. Device users made up the majority (72.6%, *n* = 98) against 27.4% (*n* = 37) non-users, and enrollment clustered in scientific fields (65.2%), followed by the humanities (28.1%) and applied medical sciences (6.7%).

### 2.3. Instrument

Participation was measured with an Arabic adaptation of the Classroom Participation Questionnaire (CPQ; Antia et al., 2007), an instrument that in its original 28-item form gauges classroom engagement along cognitive and affective lines. The authors have permission to use this instrument from the copyright holders (Antia et al., 2007; Stinson, 2026). The survey administered to participants comprised three sections: an opening section that introduced the study and its purpose, a second section that gathered demographic information (gender, degree of hearing loss, hearing-device use, and field of study), and a third section that contained the classroom-participation items described here. Adaptation for this research program followed recognized procedures for cross-cultural translation (Beaton et al., 2000; International Test Commission, 2017): two bilingual specialists in education and disability independently translated the items into Arabic, and their versions were synthesized into a single preliminary form through collaborative review; two further bilingual reviewers, who had not seen the original English version, back-translated the Arabic form into English, and discrepancies were reviewed and resolved to preserve semantic equivalence. The resulting version was then evaluated by a panel of four experts in d/Dhh education, all proficient in Arabic and English, who assessed the clarity, cultural appropriateness, and contextual fit of each item for higher education, and minor refinements were introduced on their feedback. During adaptation, wording that referred to school settings was revised for the university context (for example, references to the teacher were reworded to refer to the university professor), and two items were removed because their content did not align with the nature of university classrooms and instructional routines, yielding a 26-item version. The adaptation preserved the four CPQ dimensions: grasp of the instructor (6 items), grasp of peers (5 items), positive affect (6 items), and negative affect (9 items). Respondents rated each item on a five-point agreement scale anchored at 1 (strongly disagree) and 5 (strongly agree), and every dimension was scored as the mean of its items. For the analyses that follow, the two cognitive dimensions, closely correlated at *r* = 0.83, were merged into a single composite index of classroom communication understanding, computed as the mean of the two subscale scores, while positive and negative affect were kept as separate outcomes.

### 2.4. Procedure and Ethical Considerations

Ethical clearance for the research was granted by the Standing Committee of Bioethics Research at Prince Sattam bin Abdulaziz University under Protocol No. SCBR-692/2026, and the work proceeded under the National Implementing Regulations of the Law of Ethics of Research on Living Creatures in the Kingdom of Saudi Arabia, with participants’ autonomy, privacy, and confidentiality safeguarded throughout. Recruitment and data collection took place between June 2026 and July 2026. To reach respondents, the team worked with coordinators of university programs serving d/Dhh students nationwide, who presented the study to eligible students and circulated an online version of the questionnaire on the team’s behalf; routing distribution in this way let students respond at their own convenience and without contacting the researchers directly. Each respondent was told that taking part was voluntary and that the data would serve research purposes alone, with anonymity and confidentiality assured and no identifying details collected. All participants provided informed consent electronically: the opening section of the online survey described the study, its purpose, the voluntary nature of participation, and the anonymity of responses, and stated that completing the questionnaire constituted consent to the use of the data; no names were collected.

### 2.5. Analytic Plan

Analyses proceeded in five stages. First, the internal structure of the adapted instrument was evaluated: sampling adequacy (Kaiser–Meyer–Olkin) and Bartlett’s test of sphericity were examined, followed by maximum-likelihood exploratory factor analysis with oblimin rotation and confirmatory factor analysis comparing the four-factor structure with a more parsimonious three-factor alternative. Second, descriptive statistics, internal-consistency reliability (Cronbach’s α) interpreted in line with established criteria for measurement quality, and distributional indices were computed for all dimensions. Third, zero-order Pearson correlations were estimated among the four dimensions to establish the bivariate structure and to verify the independence of the two affective systems. Fourth, two parallel multiple regression models estimated positive and negative emotional experience, respectively, from communication understanding, with gender and hearing-device use entered as covariates; standardized coefficients and bias-corrected bootstrap confidence intervals (5000 resamples) were reported. Fifth, the central differential-association hypothesis was tested using the z test for the difference between two dependent, overlapping correlations (Meng et al., 1992) (the correlation of understanding with positive affect versus with negative affect). Gender differences in each affective dimension were further characterized using independent-samples t tests and Cohen’s d. Analyses were conducted at *α* = 0.05 (two-tailed). Common-method variance was probed with Harman’s single-factor test. No correction for multiple comparisons was applied across the reported tests; the attendant risk of Type I error inflation is acknowledged in the Limitations. Because the design is cross-sectional and correlational, all reported associations are interpreted in terms of explained variance rather than causal or temporal relationships between variables.

## 3. Results

### 3.1. Internal Structure and Reliability of the Arabic CPQ

Evidence for the validity of the adapted instrument was established during the adaptation process. Content validity was supported through expert-panel review, in which specialists in special education and psychometrics evaluated the relevance, clarity, and cultural appropriateness of each item and guided the refinement and removal of items prior to administration. The instrument also retained the four theoretically specified dimensions of the original questionnaire (grasp of the instructor, grasp of peers, positive affect, and negative affect), consistent with its intended structure; the strong correlation between the two cognitive dimensions (*r* = 0.83) further supported combining them into a single composite index of communication understanding.

Structural validity was examined through factor analysis of the 26 items. The data were well suited to factoring, Kaiser–Meyer–Olkin = 0.90 and Bartlett’s test of sphericity, χ^2^(325) = 3165, *p* < 0.001. An exploratory factor analysis (maximum-likelihood extraction, oblimin rotation) recovered factors corresponding to the theorized dimensions of the CPQ: the positive-affect and the negative-affect items each formed a clear, well-separated factor, whereas the instructor- and peer-understanding items were highly interrelated and less cleanly distinguished, consistent with the strong correlation between them (*r* = 0.83) and supporting their combination into a single communication-understanding composite for the substantive analyses. A confirmatory factor analysis of the four-factor model indicated acceptable, if not optimal, fit for a newly adapted instrument, χ^2^(293) = 607.18, χ^2^/df = 2.07, *CFI* = 0.90, *TLI* = 0.89, *RMSEA* = 0.089; this model fit better than a three-factor model that merged the two understanding dimensions (*CFI* = 0.88, *RMSEA* = 0.097). Together with the reliability evidence reported below, these results support the internal structure of the Arabic CPQ in this university sample, while indicating that the two understanding subscales function largely as one dimension. Harman’s single-factor test indicated that the first unrotated factor accounted for 41.9% of the total variance, below the conventional 50% threshold, suggesting that common-method variance is unlikely to account for the observed pattern of associations.

Reliability was evaluated through internal consistency. In the present sample, Cronbach’s alpha was excellent for all dimensions: grasp of the instructor (α = 0.94), grasp of peers (α = 0.91), positive affect (α = 0.91), and negative affect (α = 0.95), with the combined cognitive composite reaching α = 0.96. These values indicate that the adapted instrument provided reliable measurement of each dimension in this population.

### 3.2. Descriptive Statistics and Reliability

As presented in Table 2, all four dimensions demonstrated excellent internal consistency (α = 0.91–0.95). On average, participants scored toward the upper end on communication understanding (*M* = 3.89, *SD* = 0.89) and on positive emotional experience (*M* = 3.91, *SD* = 1.15), whereas negative emotional experience sat closer to the midpoint of the scale (*M* = 2.72, *SD* = 1.21). Distributional indices were within conventionally acceptable limits for parametric analysis.

### 3.3. Bivariate Structure

Zero-order correlations are reported in Table 3. Communication understanding was strongly and positively correlated with positive emotional experience (*r* = 0.69, *p* < 0.001), consistent with H1, but only weakly with negative emotional experience (*r* = 0.24, *p* = 0.006). Critically, the positive and negative affective dimensions were not significantly correlated with one another (*r* = 0.16, *p* = 0.068), supporting their treatment as empirically independent systems rather than opposite poles of a single dimension. To examine whether this association merely reflects the item overlap noted earlier, it was recomputed after excluding the two positive-affect items that name a communication partner (items 13 and 14, which concern comfort when talking with other students and with the course instructor): the correlation was essentially unchanged (*r* = 0.69, *p* < 0.001), and when positive affect was restricted to the three group-discussion feeling items that contain no comprehension content (*α* = 0.86), the association remained strong (*r* = 0.66, *p* < 0.001). The understanding–positive affect link is therefore not an artifact of overlapping item wording.

### 3.4. Associations of Communication Understanding with Positive and Negative Affect

Two parallel regression models are summarized in Table 4. In the model estimating positive emotional experience, communication understanding was a strong correlate (*β* = 0.68, 95% bootstrap CI [0.56, 0.77], *p* < 0.001), and the model accounted for 53% of the variance, *F*(3, 131) = 48.99, *p* < 0.001. In the model estimating negative emotional experience, communication understanding was a significant but substantially weaker correlate (*β* = 0.27, 95% bootstrap CI [0.06, 0.43], *p* < 0.01), and the model accounted for only 11% of the variance, *F*(3, 131) = 5.32, *p* = 0.002. A test for the difference between these two dependent, overlapping correlations confirmed that understanding was associated with positive experience significantly more strongly than with negative experience, *z* = 5.05, *p* < 0.001 (Meng et al., 1992), supporting H2.

### 3.5. Independent Contributions of Gender and Device Use

Gender contributed as an independent additive correlate in both models. Controlling for communication understanding, female students reported higher positive experience (*β* = 0.42, *p* < 0.001) and lower negative experience (*β* = −0.40, *p* = 0.02) than male students. Descriptively, the gender contrast was moderate for positive experience (female *M* = 4.26, *SD* = 1.03; male *M* = 3.72, *SD* = 1.17; *t*(133) = 2.73, *p* = 0.007, *d* = 0.49) and for negative experience (female *M* = 2.40, *SD* = 1.12; male *M* = 2.90, *SD* = 1.23; *t*(133) = −2.35, *p* = 0.020, *d* = −0.42). Hearing-device use did not contribute significantly to either model once communication understanding was accounted for (positive: *β* = 0.12, *p* = 0.39; negative: *β* = −0.23, *p* = 0.24), indicating that hearing-device use added no independent explanatory variance beyond communication understanding. H3 was therefore partially supported: gender contributed beyond communication understanding, whereas hearing-device use did not.

## 4. Discussion

This study asked how comprehension of classroom communication relates to the positive and negative dimensions of communication-related affect among d/Dhh university students, using an Arabic adaptation of the CPQ. Communication understanding was strongly associated with positive affect and only weakly with negative affect, and the two affective dimensions were largely independent. As discussed below, part of the strong positive association reflects the shared content of the understanding and positive-affect items, a feature already visible in the original validation of the instrument. The sections that follow interpret each association in turn and situate the findings within the existing literature on d/Dhh learners.

### 4.1. Communication Access and Positive Affect

The strong association between communication understanding and positive affect is consistent with the idea that being able to follow classroom talk supports the pleasant, engaged feelings that accompany participation. It must be noted, however, that the understanding and positive-affect items overlap in content, both referring to the experience of communicating successfully in class; the magnitude of this association therefore reflects shared measurement content as much as a substantive relationship. This reading aligns with the multidimensional view of participation set out by Antia et al. (2007) and with evidence that accessible, responsive classroom settings are associated with belonging and positive engagement among d/Dhh students (Antia et al., 2011; Hintermair, 2008).

### 4.2. Communication Access and Negative Affect

The weak association between communication understanding and negative emotional experience is, on its face, counterintuitive, since one might assume that students who understand more would feel less frustration, anxiety, and isolation. The results indicate otherwise, and the bivariate model of affect supplies a coherent explanation. If positive and negative affect are governed by partially separate systems (Cacioppo & Berntson, 1994; Watson & Tellegen, 1985), then improving comprehension can raise the positive system without necessarily lowering the negative one. The negative dimension appears to be sustained by influences that lie beyond the reach of comprehension, above all the social and identity-related pressures that d/Dhh students continue to meet. Research on deaf adolescents shows that perceived discrimination erodes subjective well-being and that deaf identity plays a pivotal part in how such experiences are processed (Ma et al., 2022), while work on psychological resilience indicates that students’ capacity to withstand distress rests on factors only loosely tied to instructional access (Xu et al., 2025). Qualitative accounts likewise reveal that even mainstreamed d/Dhh students who manage to follow lessons may still contend with social exclusion and the strain of coping with communication barriers (Eichengreen et al., 2026), and that adjustment to the university learning environment is shaped by psychosocial as much as instructional conditions (Percival et al., 2025). Taken together with the present findings, this body of work implies that frustration and isolation are not simply the mirror image of weak comprehension; they carry their own antecedents and will not necessarily recede as access improves. The reliability of the asymmetry, confirmed by a direct test of the difference between the two associations, gives empirical weight to this distinction and warns against treating emotional well-being as a uniform by-product of accessibility. It should be underlined that the identity-related and psychosocial influences invoked here were not measured in the present study; they are offered as theoretically grounded directions for future research rather than as tested explanations.

### 4.3. The Independent Role of Gender

Gender contributed to both affective dimensions over and above communication understanding, with female students reporting more positive and less negative emotional experience than male students. Tellingly, gender was not associated with communication understanding itself, so its bearing on emotion did not pass through comprehension. The result instead implicates social and identity-related processes as the route of the gender effect, the same family of influences identified for negative affect. Its direction fits qualitative evidence that female d/Dhh students in Saudi universities often describe comparatively positive social experiences within their academic settings (Alsamih, 2024), although the cross-sectional, self-reported character of the data warrants caution about why the difference emerges. Rather than a nuisance variable, gender here marks the fact that the affective experience of participation is patterned by social position in ways that the provision of access does not by itself reach.

### 4.4. Hearing-Device Use

Although hearing-device use was associated with communication understanding at the bivariate level, it showed no independent association with either affective dimension once understanding was taken into account. One interpretation is that device use relates to affect indirectly, by supporting comprehension, rather than directly; however, no mediation or path analysis was conducted, so this remains a tentative interpretation rather than a tested effect. What the data support directly is that, among the variables examined, perceived communication understanding rather than device use is the dimension most closely tied to communication-related affect.

### 4.5. Theoretical Contribution

Beyond its applied value, the study contributes in three ways. First, it adapts the CPQ into Arabic and extends its use from the school-aged samples with which it was developed to a university population, providing initial evidence that the adapted subscales function reliably in this new context. Second, it shows that positive and negative communication-related affect behave as largely separable dimensions in this population, consistent with bivalent models of affect. Third, it does so while making explicit the item overlap between the understanding and positive-affect subscales, so that the strong association between them is interpreted transparently rather than as a novel substantive mechanism. Interpreted within these bounds, the findings speak to the emotional texture of classroom communication rather than to emotional well-being at large: they identify which affective experiences travel with communication access and which do not, and they thereby offer a tractable entry point for future work on how everyday communicative episodes feed into d/Dhh students’ inclusion, belonging, and sustained participation in higher education, questions that require measures broader than the CPQ.

### 4.6. Limitations

Several limitations bound these conclusions. The design is cross-sectional, so although the analyses are framed in directional terms consistent with the hypothesized model, they cannot establish causation; the possibilities that emotional experience colors perceived understanding, or that a third factor drives both, cannot be excluded. Accordingly, no causal inferences can be drawn from these data. The sample, recruited by convenience and modest in size, limited the power available for testing interaction and moderation effects and made latent-variable structural modeling inadvisable, which is why observed-variable regression was preferred (a full latent structural model for these data would estimate well over sixty free parameters, pushing the case-to-parameter ratio far below recommended minima). Some characteristics of theoretical interest, including the availability of sign-language interpretation and field of study, were too unevenly distributed to support stable comparison and were left unexamined; for the same reason they could not be entered as covariates in the regression models. All measures were self-reported, raising the prospect of common-method and social-desirability bias, and although Harman’s single-factor test suggested that common-method variance was not the dominant influence, shared method effects cannot be fully excluded; and perceived understanding may not correspond exactly to objective comprehension. Finally, the sample was confined to Saudi public universities, so extension to other systems and cultural contexts should proceed with care, and generalization to private higher-education institutions or to other Arab regions in particular should be made with caution. In addition, no adjustment for multiple comparisons was applied, so some risk of Type I error inflation across the reported tests remains. Three further limitations follow from the measure itself. First, the understanding and positive-affect subscales share communication-specific content, so the strong association between them partly reflects item overlap rather than a relationship between fully distinct constructs. Second, the CPQ affect subscales capture communication-related affect rather than the broader achievement emotions described by control-value theory, so the findings speak to feelings about communicating in class rather than to academic emotion in general. Third, although the internal structure of the adapted instrument was examined through exploratory and confirmatory factor analyses, no mediation or path model was estimated, so statements about how the variables are ordered remain interpretive.

### 4.7. Implications and Future Research

The pattern documented here has direct implications for how Saudi universities pursue inclusion under Vision 2030. Because positive emotional experience is so closely tied to communication access, institutions can raise the positive engagement of d/Dhh students through concrete, scalable investments in accessibility: providing real-time captioning and note-taking, equipping faculty with inclusive communication and instructional-design practices, and integrating emerging AI-enabled tools that widen access to spoken classroom discourse (Alkahtani, 2024; Alkharji & Loh, 2022). Because the cognitive dimensions of understanding the instructor and peers were the strongest correlates of positive experience, accessibility efforts that target both formal instruction and informal peer interaction are likely to yield the greatest affective benefit.

At the same time, the weak link between access and negative emotional experience indicates that accessibility provision cannot, on its own, relieve the distress, frustration, and isolation that some d/Dhh students continue to feel. Reducing the negative dimension calls for a separate and complementary line of support directed at belonging, social connection, peer awareness, identity, and psychological well-being, delivered through counseling services, peer-mentoring and deaf-community networks, and faculty and peer awareness initiatives. The central practical message is therefore a two-track model: accessibility and psychosocial support address distinct, non-substitutable needs, and treating one as a proxy for the other risks leaving a substantial part of students’ emotional experience unaddressed.

These conclusions also chart a clear agenda for future research. Longitudinal designs are needed to clarify the temporal ordering suggested by these associations and to test whether gains in communication access precede gains in positive emotion. Future studies should measure the proposed social and identity-related antecedents of negative emotion directly, including perceived discrimination, stigma, deaf identity, and sense of belonging, so that the mechanism inferred here can be tested rather than assumed. Mixed-methods work that pairs survey data with students’ own narratives could illuminate why comprehension and distress diverge, while multi-institution and cross-national samples would establish how far the asymmetry generalizes beyond Saudi public universities. Finally, intervention research contrasting access-focused with psychosocially focused support would provide the most direct test of the two-track model and offer practical guidance for resource allocation. Finally, the psychometric evidence reported here should be read as an initial validation of the Arabic CPQ in this population: confirmation in independent samples, together with formal measurement-invariance testing across gender and language versions and, where sample sizes permit, fully latent structural models, remains a priority for future research.

## 5. Conclusions

Among d/Dhh university students in Saudi Arabia, perceived access to classroom communication was strongly associated with communication-related positive affect and only weakly with negative affect, and the two affective dimensions were largely independent. Part of the strong positive association reflects the shared content of the understanding and positive-affect subscales, so it should be read with the instrument’s structure in mind rather than as a distinct causal mechanism. Interpreted in this light, the study extends an established measure to a new language and a university population and clarifies how communication access relates to how d/Dhh students feel about communicating in class, while pointing to social and identity-related factors, beyond comprehension, as the likely drivers of negative affect.

## Figures and Tables

**Table 1:** Sample Characteristics.

Characteristic	*n*	%
Gender		
Male	86	63.7
Female	49	36.3
Degree of hearing loss		
Severe	89	65.9
Moderate	36	26.7
Mild	10	7.4
Hearing-device use		
Uses a device	98	72.6
Does not use a device	37	27.4
Field of specialization		
Scientific disciplines	88	65.2
Humanities	38	28.1
Applied medical sciences	9	6.7

**Table 2:** Descriptive Statistics and Internal Consistency for Study Dimensions.

Dimension	*k*	*M*	*SD*	α	Skew
Understanding instructor	6	3.93	0.95	0.94	−1.30
Understanding peers	5	3.84	0.90	0.91	−0.97
Communication understanding (composite)	11	3.89	0.89	0.96	
Positive emotional experience	6	3.91	1.15	0.91	−0.80
Negative emotional experience	9	2.72	1.21	0.95	0.43

Note. *k* = number of items. The cognitive composite combines the understanding-instructor and understanding-peers items.

**Table 3:** Zero-Order Correlations Among Study Dimensions.

Dimension	1	2	3	4
1. Understanding instructor	1.00			
2. Understanding peers	0.83 ***	1.00		
3. Positive emotional experience	0.67 ***	0.66 ***	1.00	
4. Negative emotional experience	0.18 *	0.28 **	0.16	1.00

Note. N = 135. * *p* < 0.05. ** *p* < 0.01. *** *p* < 0.001.

**Table 4:** Parallel Regression Models for Positive and Negative Emotional Experience.

Variable	β	*SE*	95% CI	*p*
Positive Emotional Experience (*R*^2^ = 0.53)				
Communication understanding	0.68	0.06	[0.56, 0.77]	<0.001
Gender (female)	0.42	0.13	[0.18, 0.67]	<0.001
Hearing-device use	0.12	0.14	[−0.16, 0.39]	0.39
Negative Emotional Experience (*R*^2^ = 0.11)				
Communication understanding	0.27	0.08	[0.06, 0.43]	0.002
Gender (female)	−0.40	0.17	[−0.75, −0.06]	0.020
Hearing-device use	−0.23	0.19	[−0.60, 0.15]	0.24

Note. Standardized coefficients (β) with bias-corrected bootstrap 95% confidence intervals (5000 resamples). The difference between the standardized coefficients for communication understanding across the two models was significant, z = 5.05, *p* < 0.001 (Meng et al., 1992).

## Data Availability

The data presented in this study are available on request from the corresponding author due to privacy and ethical restrictions concerning a small, potentially identifiable participant population.
